# The Management of Iatrogenic Ureterovaginal Fistula in a Resource-Limited Setting: A 12-Year Experience at Four Fistula Surgery Centers in Uganda

**DOI:** 10.7759/cureus.76170

**Published:** 2024-12-21

**Authors:** Rogers Kajabwangu, Verena Geissbüehler, Leevan Tibaijuka, Onesmus Byamukama, Paul K Kalyebara, Brenda Ainomugisha, Thomas Margolis, Henry Lukabwe, Joseph Njagi, Henry M Lugobe, Musa Kayondo

**Affiliations:** 1 Obstetrics and Gynecology, Mbarara University of Science and Technology, Mbarara, UGA; 2 Obstetrics and Gynecology, University of Basel, Geneva, CHE; 3 Obstetrics and Gynecology, University of California, Los Angeles, USA; 4 Obstetrics and Gynecology, Kisiizi Hospital, Mbarara, UGA

**Keywords:** iatrogenic, reimplantation, resource-limited setting, ureteric injury, ureterovaginal fistula

## Abstract

Background

Ureterovaginal fistulae usually follow iatrogenic injury to the ureter during pelvic surgery. This manifests as urine incontinence and results in serious psychosocial effects on women. Ureterovaginal fistulae unlike vesicovaginal fistulae present challenges in diagnosis and management especially in resource-constrained settings.

Objective

The objective of this study is to describe the magnitude, etiology, diagnosis, management, and outcomes of iatrogenic ureterovaginal fistula in Uganda over a 12-year period.

Methods

A retrospective review of charts for women who had fistula repair at four fistula repair centers in Uganda from 2010 to 2021 was conducted. The diagnosis of ureterovaginal fistula was made clinically using a history of leakage of urine through the vagina following a pelvic surgery, a negative methylene blue dye test, and a three-swab test. All women were managed using open transvesical ureteral reimplantation with or without a Boari flap. The outcome of surgery was successful fistula repair with urine continence and was determined at two months post-surgery.

Results

Overall, 477 women were managed for genitourinary fistulae during the study period. Approximately one in every 10 women with genitourinary fistula had an iatrogenic ureterovaginal fistula (n=47, 9.8%). The mean age of women with ureterovaginal fistula was 31.9 (SD: ±11.8) years. The majority of ureterovaginal fistulae (n=33, 70.7%) followed cesarean sections done at general hospitals (n=22, 46.8%) by medical officers (n=32, 68.1%). Clinical assessment was accurate in diagnosing ureterovaginal fistula. Successful fistula repair was achieved in 45 (95.7%) cases.

Conclusion

Iatrogenic ureterovaginal fistulae are common in Uganda, and most follow cesarean section performed at lower-level health facilities by medical officers. In resource-limited settings where advanced diagnostic techniques are not available or not affordable, simple stepwise clinical evaluation is effective in making a diagnosis. Open ureteral reimplantation with or without a Boari flap has a high successful repair rate.

## Introduction

A ureterovaginal fistula is an abnormal communication between the ureter and the vagina that often results from unidentified accidental injury to the ureter during pelvic surgery [1]. The ureter is susceptible to injury during pelvic surgery because of its close proximity to pelvic structures such as the uterus, cervix, broad ligament, and urinary bladder [2]. Furthermore, anatomical changes during pregnancy and pelvic pathologies such as fibroids, ovarian masses, endometriosis, pelvic inflammatory disease, and adhesions from previous abdominal surgery lead to alteration in the course of the pelvic ureter making it more susceptible to injury especially if the surgeon has limited skills [3]. Mechanisms of ureteric injuries include contusion, kinking, devascularization, laceration, suture-ligation, and circumferential transection [4].

Ureterovaginal fistula is one of the sequelae of ureteric injury, the others being ureteric obstruction, the loss of kidney function, and death [5]. Ureterovaginal fistulae often present with urinary incontinence, which has a lot of devastating effects on the quality of life of the affected women in the form of mental, social, marital, and financial repercussions [6].

In high-income countries, ureteral injury is more common during gynecological surgery than obstetric procedures [7]. Open gynecological surgery procedures account for 0.5%-1.5% of the injuries, while laparoscopic surgeries account for 0.5%-14% [8]. However, this seems to be different in low- and middle-income countries where most of the injuries follow emergency obstetric surgeries with cesarean section accounting for more than 50% of cases, while hysterectomy and uterine repair for ruptured uterus account for about 27% [9]. Efforts to make cesarean section and other emergency operations more accessible in these countries have been fairly successful but have not been followed with strategies to ensure that the surgeries are safe [10]. There are still surgical training challenges coupled with human resource shortages possibly explaining the surgical errors that lead to these injuries [11].

The diagnosis and management of ureterovaginal fistula in low-resource settings are a challenge. The standard diagnosis usually involves a combination of a history of urine incontinence following pelvic surgery, dual-dye test, intravenous pyelogram (IVP), cystoscopy, and CT urogram [12-14]. These radiological tests are also important in follow-up for assessing ureteral patency post-surgery. However, these advanced tests are not readily available in public health facilities in resource-limited settings. They can only be accessed in private imaging facilities at a cost that most fistula patients cannot afford. Furthermore, the funding for the fistula camps in these facilities is not sufficient to cater for costly investigations such as IVP and CT scans, which call for the use of low-cost but equally sensitive diagnostic methods to make a diagnosis.

The treatment of ureterovaginal fistula is usually a laparotomy with ureteral reimplantation, but more complex procedures such as Boari flap, psoas hitch, ileo-ureteral interposition, and uretero-ureteral anastomosis may be performed in more difficult cases especially when there is an extensive loss of the ureteral length [15,16]. However, specialists capable of performing these complex procedures in low-resource settings are limited. Furthermore, less invasive treatment techniques such as endoscopic ureteral stenting, in addition to being unavailable, may not be applicable in this setting due to the extensive obstetric trauma preceding these injuries that cause a distortion of the pelvic anatomy [17].

There is a paucity of data on the diagnosis, management, and outcomes of ureterovaginal fistula, yet this evidence is important for advocacy in scaling up the prevention and treatment for this devastating condition in resource-limited settings where women with fistula face various challenges in accessing care [18].

Therefore, in this study, we aimed to describe the etiology, diagnosis, management, and outcomes of ureterovaginal fistula at four public fistula surgery centers in Uganda.

This article was previously presented as a meeting abstract at the 8th International Conference of the Society of Obstetric Fistula Surgeons (ISOFS) on 3 November 2022 (https://isofs-global.org/event/view/4).

## Materials and methods

Study design and study setting

This was a retrospective chart review of women who had fistula surgery at four public fistula repair centers in Uganda during fistula repair camps from 2010 to 2021. The centers included Mbarara Regional Referral Hospital in Southwestern Uganda, Lira Regional Referral Hospital in Northern Uganda, Nakaseke General Hospital in Central Uganda, and Bwindi Community Hospital in Western Uganda. These centers all use a paper-based system of record-keeping. The surgical team was led by fistula surgeons from Mbarara University of Science and Technology (MUST).

Study population and recruitment

We identified files for all the patients who had undergone urogynecological surgery. We then reviewed all files of women who were managed for genitourinary fistula at the four centers between 2010 and 2021. From these, we identified all those that had a diagnosis of ureterovaginal fistula for data collection.

Diagnosis of iatrogenic ureterovaginal fistula

The iatrogenic ureterovaginal fistula was diagnosed in women who had leakage of urine per the vagina following any pelvic surgery such as cesarean section, uterine repair for ruptured uterus, obstetric hysterectomy, and elective hysterectomy for gynecological conditions and urine pooling in the vagina with no obvious defect along the entire anterior vaginal wall on speculum examination coupled with a negative methylene blue dye test after backfilling the bladder with a minimum of 120 mL of diluted methylene blue. A three-swab test (three cotton swabs placed at different levels in the vagina) was also performed after the methylene blue test. The ureterovaginal fistula was diagnosed if none of the swabs was stained with methylene blue but all wet with urine.

Description of the diagnostic procedures

The methylene blue test was performed as the initial diagnostic examination for all patients with urinary incontinence. The dye (120 mL) was introduced into the bladder through a Foley catheter of size 16, followed by observation to see if it was leaking into the vagina. When the dye test was negative despite the pooling of urine in the vagina, a three-swab test helped the team to decide whether this could have been a ureterovaginal fistula or a small (pinhole) vesicovaginal fistula. Three separate cotton swabs, one above the other, were placed in the upper, middle, and lower vagina. The bladder was then filled with diluted methylene blue, and the swabs were removed after 10 minutes. If all three swabs were not stained with methylene blue but all became wet with urine, a diagnosis of ureterovaginal fistula was made. If all three swabs were stained blue, then a diagnosis of a pinhole high vesicovaginal fistula was made. If the lower two swabs were stained blue and the upper colorless and dry, then a small mid-vesicovaginal fistula was confirmed. If only the lower swab was stained blue and the upper two swabs remained colorless and dry, then a pinhole urethrovaginal fistula was confirmed [19].

The diagnosis of ureterovaginal fistula was then confirmed by intraoperative findings of the absence of the spillage of urine from the affected ureteral orifice on cystotomy. The standard confirmatory diagnostic tests such as intravenous pyelogram, CT pyelogram, and cystoscopy were not done because they were not readily available at these public fistula repair facilities nor affordable in the private imaging centers.

Surgical technique

The women underwent surgical repair of the ureterovaginal fistula after obtaining informed consent. All the women had a laparotomy with the exploration of the ureteric bed on both sides to identify the affected ureter. The affected side was usually the one where the ureter was dilated. A cystotomy with the exploration of the trigone and the visualization of the ureteral orifices was done to finally confirm the diagnosis of the ureterovaginal fistula and the side affected. This was done after giving the patient an intravenous bolus of 10 mg of furosemide. The affected ureter was the one that was not spilling urine through its ureteric orifice.

After identifying the affected ureter, it was mobilized from its peritoneal and broad ligament attachments up to the point of injury. The bladder was also mobilized from its pelvic attachments. This was done to minimize tension during reimplantation. The main procedure that was done was a ureteroneocystostomy (the implantation of the injured ureter into the bladder) with absorbable sutures. In cases where the ureter had been injured at a relatively high level (above the pelvic brim) resulting in a significant loss of ureteral length, a Boari flap was performed. In all cases, a ureteric stent was inserted and tied to the Foley catheter using a nonabsorbable suture (Prolene 2/0). The bladder was then closed in two layers with absorbable suture. Postoperatively, both the ureteral stent and Foley catheter were kept in for two weeks. In addition, antibiotics and other components of postoperative care such as analgesia, fluid administration, ambulation, and wound care were offered to all the patients. The women stayed in the hospital under observation until the removal of the Foley catheter and the ureteral stent. All surgeries were performed by a team of urogynecologists/fistula surgeons, as part of the routine management of genitourinary fistula in these fistula repair centers. The women were discharged and reviewed after two months.

## Results

Demographic and clinical characteristics of the participants

A total of 477 genitourinary fistulae were managed during the study period. Of these, 9.9% (47/477) had ureterovaginal fistulae, 30.6% (147/477) had vesicovaginal fistulae, 15.1% (72/477) had vesico-cervical fistulae, 37.9% (181/477) had urethrovaginal fistulae, and 6.5% (31/477) had vaginal vault fistulae. The mean age at diagnosis of the women with ureterovaginal fistula was 31.9 (SD: ±11.8) years, and the majority were of parity>4 (25/47, 53.2%; range: 1-7). Most fistulae (33/47, 70.7%) developed after a cesarean section, and a significant number (9/47, 19.5%) occurred after a hysterectomy for obstetric indications mainly ruptured uterus. Most of these antecedent surgeries (22/47, 46.8%) were performed at general hospitals and by medical officers (32/47, 68.1%) as shown in Table 1.

**Table 1:** Demographic and clinical characteristics of the patients The data has been represented as N (%)

Characteristic		N=47	
	Description	Frequency, n	Percentage
Age	<25 years	10	21.3
	25-35 years	15	31.9
	>35 years	22	46.8
Occupation	Unemployed	43	91.5
	Employed	4	8.5
Marital status	Single	5	10.6
	Married	36	76.6
	Divorced/separated/widowed	6	12.8
Parity at diagnosis	1	10	21.3
	2-4	12	25.5
	≥5	25	53.2
Duration of incontinence	<3 months	16	34.0
	3 months to one year	14	29.8
	≥1 year	17	36.2
Antecedent surgery	Cesarean section	33	70.7
	Obstetric hysterectomy	9	19.5
	Hysterectomy for gynecological indications	5	9.8
Level of facility for antecedent surgery	Referral hospital	19	40.4
	General hospital	22	46.8
	Health center IV	6	12.8
Level of training of surgeon who performed antecedent surgery	Intern doctor	7	14.9
	Medical officer	32	68.1
	Resident	4	8.5
	Specialist	4	8.5

Perioperative characteristics of the participants

The perioperative characteristics of the participants are shown in Table 2. For all the participants, the stepwise clinical assessment was used to diagnose all ureterovaginal fistulae. At surgery, the left ureter was the most affected (n=24/47, 51.1%). Most injuries to the ureter (n=45/47, 95.6%) occurred within the pelvis, while in two women, it occurred at the level of the pelvic brim around the ovarian fossa. The commonest fistula repair surgery done was ureteric reimplantation alone (45/47, 95.7%), while in a few of the patients (2/47, 4.3%), a Boari flap was performed due to a short ureter. The surgical complications encountered included intraoperative hemorrhage that required blood transfusion (5/47, 11%) of the women and postoperative wound infection (5/47, 11%). The women with wound infection all improved following daily dressing and antibiotic treatment.

**Table 2:** Perioperative characteristics of the participants (N=47) *Some participants had more than one complication

Characteristic	Description	Frequency	Percentage
Affected ureter	Left	24	51.1
	Right	12	25.5
	Bilateral	11	23.4
Level of injury	Above the pelvic brim	2	4.3
	Below the pelvic brim	45	95.7
Surgery performed	Ureteric reimplantation alone	45	95.7
	Ureteric reimplantation with a Boari flap	2	4.3
*Surgical complications	No complication	40	85.1
	Intraoperative hemorrhage	5	11.0
	Wound infection	5	11.0

Outcomes of surgery

**Figure 1:**
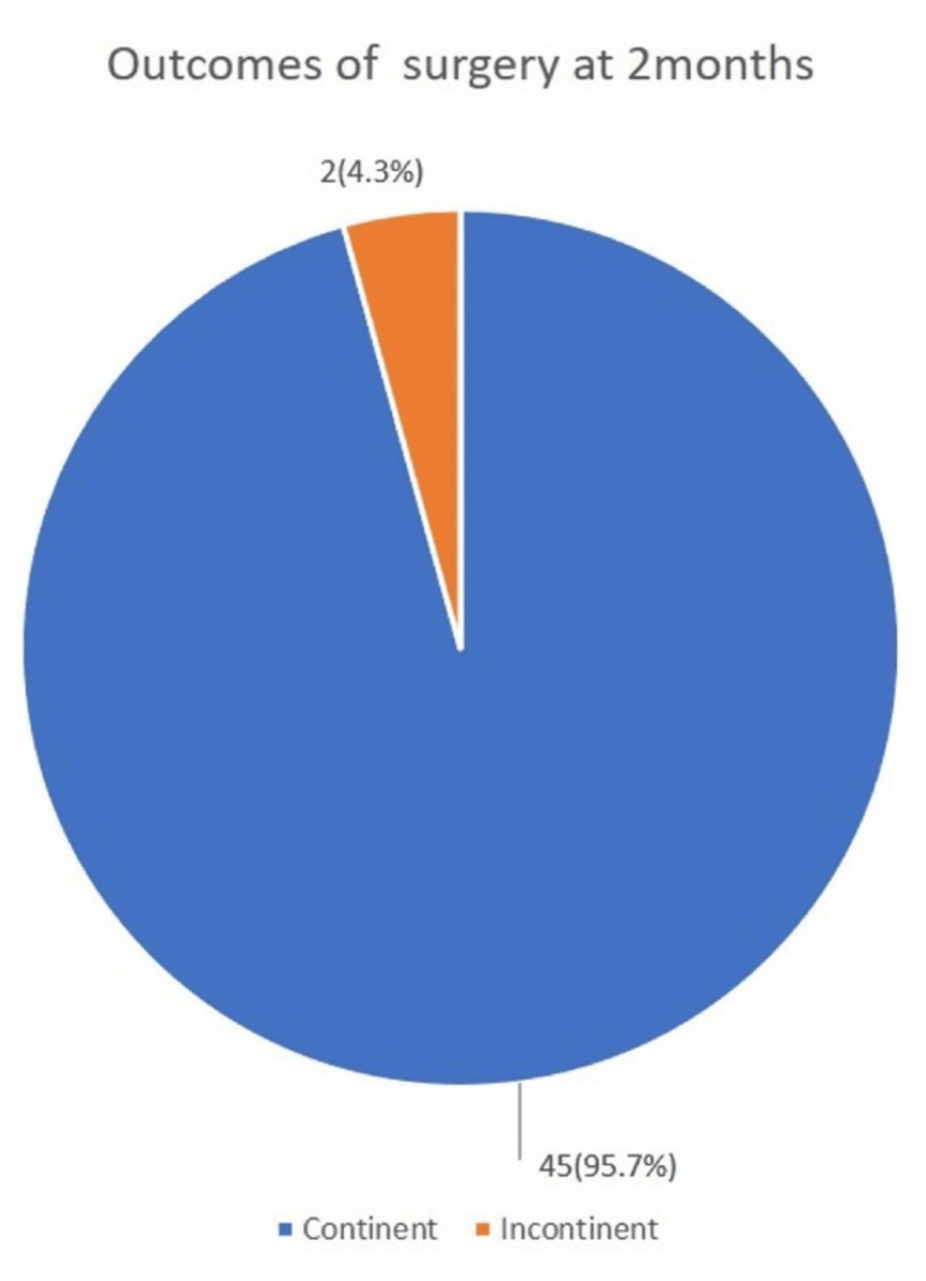
Outcomes of ureterovaginal fistula surgery

Out of the 47 women managed for ureterovaginal fistula, 45 (95.7%) had a successful closure of the fistula with continence as shown in Figure 1. There were two women with unsuccessful fistula closure. The cause of failure in these participants was due to a breakdown of the reimplantation. However, ureteric reimplantation was repeated, and in both, it was successful.

## Discussion

This study set out to describe the magnitude, etiology, diagnosis, and management of iatrogenic ureterovaginal fistula in a resource-limited setting. We found that ureterovaginal fistula is common, occurring in one in 10 women with genitourinary fistula. Ureterovaginal fistula commonly followed obstetric operations especially cesarean section performed by medical officers. A simple stepwise clinical evaluation combined with intraoperative findings is effective in diagnosing ureterovaginal fistula and locating the affected side. The success rate of open transvesical ureteral reimplantation with or without a Boari flap in this setting is 95.7%.

The magnitude of 10% is similar to that found by Shaw et al. in a study done at three Cleveland Clinics in the United States of America [19]. However, this proportion is lower than the 33.9% (273/805) found in a review done in 11 African countries by Raassen et al. [9]. As reported in this review, iatrogenic ureterovaginal fistulae were likely to occur in surgeries performed by medical officers. This is not surprising because medical officers are usually early-career clinicians with very little surgical experience who are posted to general hospitals and health center IVs to work independently. Since most of the emergency obstetric surgeries especially cesarean section in Uganda are done by medical officers, it explains the high proportion of iatrogenic ureterovaginal fistula in this study [20]. Further, the cesarean delivery rates in Uganda have increased from 8.5% in 2012 to 11% in 2016, but these have not been matched by improvements in human resource to provide safe cesarean section [21]. Surgical training challenges coupled with human resource shortages still exist possibly explaining the surgical errors that lead to these injuries [11,22].

In agreement with previous studies in low-income countries, most of the ureterovaginal fistulae in our study followed injuries sustained during obstetric surgeries particularly cesarean section [2,9] as opposed to studies done in high-income countries where the majority are due to gynecological surgeries [7,20,23]. Like in other low-income countries, obstructed labor and repeat cesarean section are the commonest indications for cesarean section in Uganda [24,25]. In prolonged obstructed labor, the fetal head gets deeply impacted in the maternal pelvis, and the lateral extension of a low transverse uterine incision may occur during delivery resulting in excessive hemorrhage. Ureteric injury in this case usually results from desperate attempts to achieve hemostasis without the proper identification of the ureter [26,27]. In the case of repeat cesarean section, scar tissue and adhesions from prior cesarean section may distort the pelvic anatomy and alter the course of the ureter making it more likely to be injured in repeat cesarean sections [9,28].

The left ureter was affected more compared to the right, as has been found in several other studies [2,9,29]. This is because the left ureter is slightly nearer to the cervix compared to the right and is also obscured by the sigmoid mesocolon [9,30].

In this study, the diagnosis of ureterovaginal fistula was made from a simple stepwise clinical evaluation, which involved a combination of a history of urine incontinence following a pelvic surgery, the absence of visible defect on speculum examination, negative methylene blue dye test, and three-swab test. A similar method was used in a rural hospital with limited resources in Nigeria [31]. This is in contrast to complex and costly methods used in high-income countries that involve the use of dual-dye test, intravenous pyelogram, cystoscopy, and CT urogram on top of the clinical evaluation [13,14,28]. However, these investigative techniques are neither available nor affordable in low-income settings [31]. In these settings, we opine that clinical assessment can be used for the diagnosis of ureterovaginal fistulae. However, clinical diagnosis alone does not provide some of the pre-operative information such as the localization of the affected ureter and the level of injury. Efforts to increase the availability of radiological investigations such as intravenous pyelography in resource-limited settings should be reenforced.

In contrast to Randawa et al., we did not do an abdominal ultrasound to evaluate the ureter and kidneys for hydroureter and/or hydronephrosis in order to determine the affected side [31]. This is because the ultrasound may be misleading since the absence of hydronephrosis does not necessarily imply that the particular side is not affected especially if the leakage from the injured ureter occurred immediately after the obstruction or if the ureter was just transected [32]. We therefore identified the affected side intraoperatively by opening the bladder and visualizing the ureteric orifices for urine spillage.

Our surgical technique of laparotomy with ureteral reimplantation with or without a Boari flap was effective in achieving continence. This is not different from findings in other similar studies, implying that this method can effectively be used in the repair of ureterovaginal fistulae in properly selected patients especially in distal ureteral injuries [2,3,33]. In more proximal injuries where the ureteral length is inadequate, a Boari flap can be used to ease tension on the anastomosis between the ureter and bladder [34]. However, newer and less invasive techniques of managing ureterovaginal fistula employed in high-income countries such as endoscopic ureteral stenting were not used in this study. This is because the equipment used in these methods is not readily available and surgeons with the skills to perform them are few.

This study describes the management of ureterovaginal fistula in a setting with limited laboratory and radiological diagnostic capacity. This is important for clinicians in low-income countries where advanced diagnostic and treatment modalities are not readily available.

Our study had some limitations: the method of clinical diagnosis without imaging may have some false-positive cases of ureterovaginal fistula leading to abandoning the surgical procedure. However, we did not encounter such cases in this study. Pre-operative renal function tests to assess for renal impairment following prolonged ureteric obstruction were not done. We were also not able to assess for postoperative ureteric strictures and renal impairment, which can be detected by investigations such as IVP and CT urogram.

## Conclusions

Iatrogenic ureterovaginal fistulae are common in our setting, and most follow cesarean section done at lower health facilities by medical officers. Simple but careful stepwise clinical evaluation is effective in diagnosing ureterovaginal fistula where investigative techniques are not available. However, imaging is still very important in diagnosis and postoperative follow-up. Open transvesical ureteral reimplantation with or without a Boari flap is highly effective in treating ureterovaginal fistula. We recommend that medical doctors conducting cesarean section and other pelvic surgeries should receive continuous training in performing safe surgery essential to reduce the burden of ureteral injuries and pre-/postoperative imaging to be included in fistula repair programs.
